# Aneurismal subarachnoid hemorrhage during the COVID-19 outbreak in a Hub and Spoke system: observational multicenter cohort study in Lombardy, Italy

**DOI:** 10.1007/s00701-021-05013-9

**Published:** 2021-10-25

**Authors:** Alessandro Fiorindi, Marika Vezzoli, Francesco Doglietto, Luca Zanin, Giorgio Saraceno, Edoardo Agosti, Antonio Barbieri, Silvio Bellocchi, Claudio Bernucci, Daniele Bongetta, Andrea Cardia, Emanuele Costi, Marcello Egidi, Antonio Fioravanti, Roberto Gasparotti, Carlo Giussani, Gianluca Grimod, Nicola Latronico, Davide Locatelli, Dikran Mardighian, Giovanni Nodari, Jacopo Carlo Poli, Frank Rasulo, Elena Roca, Giovanni Marco Sicuri, Giannantonio Spena, Roberto Stefini, Oscar Vivaldi, Cesare Zoia, Stefano Calza, Marco Maria Fontanella, Marco Cenzato

**Affiliations:** 1 Neurosurgery, Spedali Civili, Department of Medical and Surgical Specialties, Radiological Sciences and Public Health, University of Brescia, P.le Spedali Civili, 1–25123 Brescia, Italy; 2 Biostatistics, Department of Molecular and Translational Medicine, University of Brescia, Brescia, Italy; 3 Neurosurgery, ASST Santi Paolo e Carlo, San Carlo Borromeo Hospital, Milan, Italy; 4 Neurosurgery, Sant’Anna Hospital, Como, Italy; 5 Neurosurgery, ASST Papa Giovanni XXIII Hospital, Bergamo, Italy; 6 Neurosurgery, Ospedale Fatebenefratelli e Oftalmico, ASST Fatebenefratelli-Sacco, Milan, Italy; 7 Neurosurgery, Humanitas Clinical and Research Center–IRRCS, Rozzano, Milan Italy; 8 Neurosurgery, Cremona Hospital, Cremona, Italy; 9 Neuroradiology, Spedali Civili, University of Brescia, Brescia, Italy; 10 Neurosurgery, San Gerardo Hospital, Monza, Università Milano-Bicocca, Milan, Italy; 11 Neurosurgery, ASST Lecco, A. Manzoni Hospital, Lecco, Italy; 12 NeuroAnesthesia and ICU, Department of Medical and Surgical Specialties, Radiological Sciences and Public Health, Brescia, Italy; 13 Neurosurgery, Ospedale di Circolo, University of Insubria, Varese, Italy; 14 Neurosurgery, Fondazione Poliambulanza Hospital, Brescia, Italy; 15 Neurosurgery, Legnano Hospital, Legnano, Italy; 16 Neurosurgery, Fondazione IRCCS Policlinico San Matteo, Pavia, Italy; 17 Neurosurgery, Grande Ospedale Metropolitano di Niguarda, Milan, Italy

**Keywords:** Intracranial bleeding aneurysm, Subarachnoid hemorrhage, COVID-19, Logistic regression, Pandemic, Hub and Spoke

## Abstract

**Background:**

Lombardy was the most affected Italian region by the first phase of the COVID-19 pandemic and underwent urgent reorganization for the management of emergencies, including subarachnoid hemorrhage from a ruptured cerebral aneurysm (aSAH). The aim of the study was to define demographics, clinical, and therapeutic features of aSAH during the COVID-19 outbreak and compare these with a historical cohort.

**Methods:**

In this observational multicenter cohort study, patients aged 18 years or older, who were diagnosed with aSAH at the participating centers in Lombardy from March 9 to May 10, 2020, were included (COVID-19 group). In order to minimize bias related to possible SAH seasonality, the control group was composed of patients diagnosed with aSAH from March 9 to May 10 of the three previous years, 2017–2018-2019 (pre-pandemic group). Twenty-three demographic, clinical, and therapeutic features were collected. Statistical analysis was performed.

**Results:**

Seventy-two patients during the COVID-19 period and 179 in the control group were enrolled at 14 centers. Only 4 patients were positive for SARS-CoV-2. The “diagnostic delay” was significantly increased (+ 68%) in the COVID-19 group vs. pre-pandemic (1.06 vs. 0.63 days, respectively, *p*-value = 0.030), while “therapeutic delay” did not differ significantly between the two periods (0.89 vs. 0.74 days, *p*-value = 0.183). Patients with poor outcome (GOS at discharge from 1 to 3) were higher during the COVID-19 period (54.2%) compared to pre-pandemic (40.2%, *p* = 0.044). In logistic regression analysis, in which outcome was the dichotomized Glasgow Outcome Scale (GOS), five variables showed *p*-values < 0.05: age at admission, WFNS grade, treatment (none), days in ICU, and ischemia.

**Conclusions:**

We documented a significantly increased “diagnostic delay” for subarachnoid hemorrhages during the first COVID-19 outbreak in Lombardy. However, despite the dramatic situation that the healthcare system was experiencing, the Lombardy regional reorganization model, which allowed centralization of neurosurgical emergencies such as SAHs, avoided a “therapeutic delay” and led to results overall comparable to the control period.

## Introduction

Subarachnoid hemorrhage from a ruptured cerebral aneurysm (aSAH) is a time-dependent disease and a neurosurgical emergency. Some authors have reported an overall decrease in the number of cases admitted for aSAH during the first phase of the SARS-CoV-2 pandemic [3, 6, 12], while others have not documented any difference in the incidence of hemorrhagic cerebrovascular disease due to vascular malformations [11] or an increase of patients admitted with a diagnosis of SAH during the COVID-19 period [35]. Furthermore, as the increased surgical risk associated with COVID-19 has become evident [14, 26], some have reported a shift towards endovascular treatment of aSAH [16].

During the first phase of the COVID-19 pandemic, the Lombardy region, which is the most populated region in Italy with nearly 10 million inhabitants, was the most affected. The Lombard Regional Council organized an emergency network during the national lockdown, creating an unprecedented Hub and Spoke system: treatment of time-dependent emergency was centralized in Hub hospitals and COVID-19 patients were treated in different satellite reinforced Spoke hospitals. All deferrable elective activities were suspended. As far as neurosurgery is concerned, three Hub centers were identified, each covering a “macro-zone” with a catchment area of more than 3 million people: Milan and hinterland (Grande Ospedale Niguarda, Milan), north and central Lombardy (Ospedale di Circolo, Varese), and south and east Lombardy (Spedali Civili, Brescia) [18, 39]. Most of the other Neurosurgery Units of the region were converted into COVID wards. All patients diagnosed with SAH with CT scan were hospitalized, in emergency, in that same hospital if it was a Hub, or immediately referred to the Hub if diagnosis was made in a Spoke hospital. To the best of our knowledge, there is no report on the efficacy of the Hub and Spoke system for aSAH during COVID-19 pandemic.

As defining the epidemiological characteristics, management, treatment, and outcomes of aSAH in Lombardy during the COVID-19 outbreak has important scientific relevance and possible practical implications, this observational multicenter study was carried out. The main endpoint of the study was evaluation of whether the COVID-19 pandemic influenced management and outcomes of aSAH compared to the pre-pandemic period. Secondary endpoints included delay in diagnosis or treatment and differences in type of treatment between the two periods.

## Materials and methods

### Study protocol and data collection

This was an observational multicenter cohort study with a control group in a 1:3 ratio. Patients aged 18 years or older, who were diagnosed with aSAH at the participating centers in Lombardy from March 9 to May 10, 2020 (corresponding to the national lockdown period due to COVID-19 pandemic, which led to the regional reorganization of neurosurgical units), were included (COVID-19 group). All patients underwent screening for SARS-CoV-2 with reverse transcriptase–polymerase chain reaction assay in a nasopharyngeal swab. Chest radiography and/or chest computed tomography (CT) scan were performed to investigate any pulmonary abnormalities.

In order to minimize the bias related to possible SAH seasonality, the control group was composed of patients diagnosed with aSAH from March 9 to May 10 of the three previous years, 2017–2018-2019 ( pre-pandemic group).

The following data were recorded for all patients (Table 1): age, sex, comorbidities, time of onset of symptoms, time of aSAH diagnosis and hospital admission, WNFS score, Fisher grade, aneurysm site, type and time of treatment, external ventricular drainage (EVD) positioning and duration, SAH-related complications, COVID-related complications, length of stay in Intensive Care Units (ICUs), length of stay in hospital (intended as Neurosurgery Unit), and Glasgow Outcome Scale (GOS) at discharge from Neurosurgery. Data collection was mainly retrospective for the COVID-19 period and completely retrospective for the pre-pandemic one. We calculated the “diagnostic delay,” i.e., the time between onset of SAH symptoms and diagnosis with CT scan, and the “therapeutic delay,” i.e., time between hospitalization and treatment; hospitalization has always immediately followed diagnosis. Vasospasm was diagnosed mainly with transcranial Doppler (TCD), defined as mean flow velocity (MFV) ≥ 120 cm/s and Lindegaard ratio ≥ 3 [4]; ischemia was defined on the basis of the evidence of a hypodense area on CT scan.

**Table 1:** Descriptive statistics on all variables (i) stratified for period (COVID-19 vs. pre-pandemic) and (ii) total sample (251 patients)

Variables	COVID-19 (*N* = 72)	Pre-pandemic (*N* = 179)	Total (*N* = 251)	*p*-value
Age at admission				0.122^**^
Mean (SD)	60.39 (13.68)	58.26 (12.87)	58.87 (13.11)	
Median (Q1, Q3)	62.00 (51.75, 71.25)	57.00 (49.50, 67.00)	59.00 (50.00, 68.00)	
Range	23.00–83.00	24.00–92.00	23.00–92.00	
Sex				0.390^*^
F	45 (62.5%)	122 (68.2%)	167 (66.5%)	
M	27 (37.5%)	57 (31.8%)	84 (33.5%)	
Smoke	7 (9.7%)	26 (14.5%)	33 (13.1%)	0.308^*^
OCPD	1 (1.4%)	5 (2.8%)	6 (2.4%)	0.510^*^
Hypertension	46 (63.9%)	67 (37.4%)	113 (45.0%)	< 0.001^*^
Diabetes	4 (5.6%)	8 (4.5%)	12 (4.8%)	0.715^*^
Obesity	6 (8.3%)	11 (6.1%)	17 (6.8%)	0.533^*^
WFNS grade				0.472^*^
1	22 (30.6%)	77 (43.0%)	99 (39.4%)	0.092^#^
2	17 (23.6%)	34 (19.0%)	51 (20.3%)	0.517^#^
3	8 (11.1%)	14 (7.8%)	22 (8.8%)	0.557^#^
4	10 (13.9%)	22 (12.3%)	32 (12.7%)	0.893^#^
5	15 (20.8%)	32 (17.9%)	47 (18.7%)	0.716
Fisher grade				0.255^*^
1	4 (5.6%)	11 (6.1%)	15 (6.0%)	0.859^#^
2	17 (23.6%)	31 (17.3%)	48 (19.1%)	0.333^#^
3	10 (13.9%)	44 (24.6%)	54 (21.5%)	0.090^#^
4	41 (56.9%)	93 (52.0%)	134 (53.4%)	0.564^#^
Diagnosis delay (in days)				0.030^**^
Mean (SD)	1.06 (2.45)	0.63 (1.99)	0.75 (2.14)	
Median (Q1, Q3)	0.00 (0.00, 1.00)	0.00 (0.00, 0.00)	0.00 (0.00, 0.00)	
Range	0.00–13.00	0.00–14.00	0.00–14.00	
Aneurysm location				0.364^*^
ACA	11 (15.3%)	17 (9.5%)	28 (11.2%)	0.274^#^
AComA	23 (31.9%)	51 (28.5%)	74 (29.5%)	0.697^#^
AICA	1 (1.4%)	0 (0.0%)	1 (0.4%)	0.637^#^
Basilar apex	5 (6.9%)	6 (3.4%)	11 (4.4%)	0.359^#^
ICA	8 (11.1%)	21 (11.7%)	29 (11.6%)	0.741^#^
MCA	12 (16.7%)	33 (18.4%)	45 (17.9%)	0.882^#^
Ophthalmic	0 (0.0%)	1 (0.6%)	1 (0.4%)	0.525^#^
PCA	0 (0.0%)	2 (1.1%)	2 (0.8%)	0.368^#^
PComA	11 (15.3%)	31 (17.3%)	42 (16.7%)	0.695^#^
PICA	1 (1.4%)	12 (6.7%)	13 (5.2%)	0.160^#^
SCA	0 (0.0%)	2 (1.1%)	2 (0.8%)	0.368^#^
VA	0 (0.0%)	3 (1.7%)	3 (1.2%)	0.269^#^
Treatment				0.701^*^
Endovascular	49 (68.1%)	125 (69.8%)	174 (69.3%)	0.783^#^
Surgical	15 (20.8%)	40 (22.3%)	55 (21.9%)	0.793^#^
None	8 (11.1%)	14 (7.8%)	22 (8.8%)	0.405^#^
Therapeutic delay (in days)				0.183^**^
No procedures	8	14	22	
Mean (SD)	0.89 (3.20)	0.74 (2.68)	0.78 (2.82)	
Median (Q1, Q3)	0.00 (0.00, 0.00)	0.00 (0.00, 1.00)	0.00 (0.00, 1.00)	
Range	0.00–17.00	0.00–29.00	0.00–29.00	
Days in ICU				0.023^**^
None	4	17	21	
Mean (SD)	8.91 (12.38)	11.32 (12.51)	10.61 (12.49)	
Median (Q1, Q3)	4.50 (0.75, 13.00)	7.00 (2.00, 16.75)	6.00 (2.00, 16.00)	
Range	0.00–57.00	0.00–77.00	0.00–77.00	
Days in hospital				0.016^**^
Mean (SD)	19.79 (12.76)	25.10 (17.31)	23.58 (16.29)	
Median (Q1, Q3)	18.00 (12.00, 24.50)	21.00 (16.00, 32.00)	20.00 (15.00, 31.00)	
Range	0.00–57.00	0.00–108.00	0.00–108.00	
EVD days				0.060^**^
No EVD	46	102	148	
Mean (SD)	19.08 (11.02)	14.83 (10.21)	15.90 (10.53)	
Median (Q1, Q3)	18.00 (10.25, 29.75)	15.00 (7.00, 21.00)	15.00 (7.00, 23.50)	
Range	3.00–38.00	1.00–48.00	1.00–48.00	
Ischemia	4 (5.6%)	38 (21.2%)	42 (16.7%)	0.003^*^
Rebleeding	6 (8.3%)	9 (5.0%)	15 (6.0%)	0.318^*^
Vasospasm	13 (18.1%)	41 (22.9%)	54 (21.5%)	0.398^*^
GOS discharge				0.016^*^
1	15 (20.8%)	26 (14.5%)	41 (16.3%)	0.221^#^
2	9 (12.5%)	8 (4.5%)	17 (6.8%)	*0.022*^#^
3	15 (20.8%)	38 (21.2%)	53 (21.1%)	0.945^#^
4	13 (18.1%)	22 (12.3%)	35 (13.9%)	0.233^#^
5	20 (27.8%)	85 (47.5%)	105 (41.8%)	*0.004*^#^
GOS discharge dummy				0.044^*^
0 (GOS discharge > 3)	33 (45.8%)	107 (59.8%)	140 (55.8%)	
1 (GOS discharge ≤ 3)	39 (54.2%)	72 (40.2%)	111 (44.2%)	
Death	15 (20.8%)	26 (14.5%)	41 (16.3%)	0.221^*^

In bold and Italics *p*-values < 0.05

^*^Chi-square test

^**^Mann–Whitney test

^#^Two proportion *z*-test on subgroups of patients

For dichotomous variables (smoke, OCPD, hypertension, diabetes, obesity, ischemia, rebleeding, vasospasm, death), the frequencies in table correspond to the *Yes* category. The *p*-values are instead computed on the $2 \times 2$ contingency table, considering also the *No* category

The study was approved by the local ethics committee (NP 4192 — SAH-COVID-LOMB). Patient consent was obtained at the time of treatment.

### Statistical analysis

The dataset contains 23 variables. For qualitative variables, absolute frequencies (%) were computed, while for quantitative variables, mean, standard deviation, median (Q1, Q3), and range (min–max) were calculated. When the descriptive statistics were stratified with respect to qualitative variables (e.g., COVID-19 vs. pre-pandemic), the chi-square test evaluated the association between couple of variables. *P*-values of the two proportions *z*-test are reported for subgroups of patients defined by the following variables: WFNS grade, Fisher grade, aneurysm location, treatment, and GOS discharge. In case of quantitative variables, the Mann–Whitney test, which identifies if two independent subsamples come from the same population, was used [9, 23, 33, 37].

Descriptive statistics are also stratified with respect to the dichotomized GOS (GOS > 3 corresponds to 0; 1 otherwise). This bivariate analysis identified a subsample of variables associated (*p*-values < 0.05) with the outcome (GOS) using them as covariates in a multivariate logistic model. The multicollinearity problem was evaluated computing the Spearman correlation coefficient between couple of quantitative variables. Output of the estimated model reports odds ratio (OR), corresponding 95% confidence interval (CI), *p*-values, pseudo *R*^2^, and AIC.

## Results

Seventy-two patients during the COVID-19 period [45 female (62.5%) vs. 27 male (37.5%)] and 179 in the control group [122 female (68.2%) vs. 57 male (31.8%)] were enrolled at 14 centers. There was no relevant differences in gender between the two groups (*p*-value = 0.390). The overall mean (SD) age was 58.87 (13.11) years: patients in the COVID-19 period were slightly older [mean (SD) is 60.39 (13.68) vs. 58.26 (12.87)] without any significant difference (*p* = 0.122). Only 4 patients were positive for SARS-CoV-2 (5.5% of subjects in COVID-19 period); hence, this variable was not considered in data analyses.

In bivariate analysis, which compared the COVID-19 vs. pre-pandemic periods (Table 1), Fisher and WFNS grades were not significantly different (*p* 0.255 and 0.472, respectively). The “diagnostic delay” was significantly increased (+ 68%) during the COVID-19 group (COVID-19 vs. pre-pandemic: 1.06 and 0.63 days, respectively; *p* = 0.030). When the “diagnostic delay” was dichotomized as “same day” vs. “at least one day,” 24 patients (33.3%) were diagnosed after at least 1 day during the COVID-19 pandemic compared to 34 patients (20.7%) in the pre-pandemic period (*p* = 0.051). “Therapeutic delay” in days did not differ significantly between the COVID-19 and pre-pandemic periods (0.89 vs. 0.74 days, *p* = 0.183).

The percentage of patients with poor outcome (GOS at discharge from 1 to 3) was higher during the COVID-19 period (54.2%) compared to pre-pandemic (40.2%, *p* = 0.044). Particularly, patients with GOS 2 were 12.5% in COVID-19 vs. 4.5% in pre-pandemic period (*p* = 0.022), while patients with GOS 5 were 27.8 in COVID-19 compared to 47.5% in pre-pandemic period (*p* = 0.004). No significant differences were seen in the type of treatment (endovascular, surgical, or no treatment) between the two groups (*p* = 0.701).

Table 2 reports descriptive statistics computed on the same variables stratified with respect to the dichotomized GOS at discharge (GOS > 3 $\Rightarrow$ 0, 1 otherwise). In this bivariate analysis, nine variables were associated with the dichotomized GOS (*p*-values < 0.05 in Table 2, last column): period (COVID-19 vs. pre-pandemic), age at admission, hypertension, WFNS grade, Fisher grade, treatment, days in ICU, ischemia, and vasospasm. These variables were used as covariates in multivariate logistic regression where the outcome was the dichotomized GOS at discharge.

**Table 2:** Descriptive statistics on all variables stratified for GOS discharge dummy

Variables	GOS discharge > 3 (*N* = 140)	GOS discharge $\le$ 3 (*N* = 111)	*p*-value
Period			0.044^*^
COVID-19	33 (23.6%)	39 (35.1%)	
Pre-pandemic	107 (76.4%)	72 (64.9%)	
Age at admission			< 0.001^**^
Mean (SD)	56.50 (12.40)	61.86 (13.43)	
Median (Q1, Q3)	55.50 (48.00, 65.00)	62.00 (54.00, 72.00)	
Range	24.00–87.00	23.00–92.00	
Sex			0.264^*^
Female	89 (63.6%)	78 (70.3%)	
Male	51 (36.4%)	33 (29.7%)	
Smoke	16 (11.4%)	17 (15.3%)	0.365^*^
OCPD	2 (1.4%)	4 (3.6%)	0.263^*^
Hypertension	52 (37.1%)	61 (55.0%)	0.005^*^
Diabetes	7 (5.0%)	5 (4.5%)	0.855^*^
Obesity	6 (4.3%)	11 (9.9%)	0.078^*^
WFNS grade			< 0.001^*^
1	76 (54.3%)	23 (20.7%)	< *0.001*^*#*^
2	35 (25.0%)	16 (14.4%)	*0.038*^*#*^
3	10 (7.1%)	12 (10.8%)	0.307^#^
4	8 (5.7%)	24 (21.6%)	< *0.001*^*#*^
5	11 (7.9%)	36 (32.4%)	< *0.001*^*#*^
Fisher grade			< 0.001^*^
1	12 (8.6%)	3 (2.7%)	0.051^#^
2	34 (24.3%)	14 (12.6%)	0.020^#^
3	35 (25.0%)	19 (17.1%)	0.131^#^
4	59 (42.1%)	75 (67.6%)	< *0.001*^*#*^
Diagnosis delay (in days)			0.343^**^
Mean (SD)	0.79 (2.30)	0.70 (1.92)	
Median (Q1, Q3)	0.00 (0.00, 1.00)	0.00 (0.00, 0.00)	
Range	0.00–14.00	0.00–13.00	
Aneurysm location			0.532^*^
ACA	16 (11.4%)	12 (10.8%)	0.877^#^
AComA	41 (29.3%)	33 (29.7%)	0.939^#^
AICA	1 (0.7%)	0 (0.0%)	0.372^#^
Basilar apex	6 (4.3%)	5 (4.5%)	0.933^#^
ICA	18 (12.9%)	11 (9.9%)	0.468^#^
MCA	21 (15.0%)	24 (21.6%)	0.174^#^
Ophthalmic	1 (0.7%)	0 (0.0%)	0.372^#^
PCA	1 (0.7%)	1 (0.9%)	0.869^#^
PComA	24 (17.1%)	18 (16.2%)	0.845^#^
PICA	9 (6.4%)	4 (3.6%)	0.316^#^
SCA	2 (1.4%)	0 (0.0%)	0.206^#^
VA	0 (0.0%)	3 (2.7%)	0.050^#^
Treatment			< 0.001^*^
Endovascular	110 (78.6%)	64 (57.7%)	< *0.001*^*#*^
Surgical	26 (18.6%)	29 (26.1%)	0.151^#^
None	4 (2.9%)	18 (16.2%)	< *0.001*^*#*^
Therapeutic delay (in days)			0.161^**^
No procedures	4	17	
Mean (SD)	0.60 (1.60)	1.04 (3.98)	
Median (Q1, Q3)	0.00 (0.00, 1.00)	0.00 (0.00, 0.00)	
Range	0.00–14.00	0.00–29.00	
Days in ICU			< 0.001^**^
None	16	5	
Mean (SD)	7.23 (9.21)	14.57 (14.55)	
Median (Q1, Q3)	3.50 (1.00, 11.00)	13.00 (3.00, 19.00)	
Range	0.00–50.00	0.00–77.00	
Days in hospital			0.180^**^
Mean (SD)	22.06 (13.83)	25.49 (18.84)	
Median (Q1, Q3)	19.00 (15.00, 25.25)	24.00 (10.50, 36.00)	
Range	2.00–108.00	0.00–94.00	
EVD days			0.450^**^
No EVD	99	49	
Mean (SD)	14.37 (8.60)	16.92 (11.58)	
Median (Q1, Q3)	15.00 (7.00, 20.00)	15.50 (6.00, 24.00)	
Range	1.00–34.00	1.00–48.00	
Ischemia	10 (7.1%)	32 (28.8%)	< 0.001^*^
Rebleeding	6 (4.3%)	9 (8.1%)	0.205^*^
Vasospasm	22 (15.7%)	32 (28.8%)	0.012^*^

In bold and Italics *p*-values < 0.05

^*^Chi-square test

^**^Mann–Whitney test

^#^Two proportion *z*-test on subgroups of patients

For dichotomous variables (smoke, OCPD, hypertension, diabetes, obesity, ischemia, rebleeding, vasospasm), the frequencies in table correspond to the *Yes* category. The *p*-values are instead computed on the $2 \times 2$ contingency table, considering also the *No* category

Before proceeding, the Spearman correlation coefficient between quantitative variables in the model (age at admission and days in ICU) was computed. Since it equals to 0.012 (*p*-value = 0.853), the collinearity problem was excluded obtaining reliable and stable estimates of regression coefficients.

The results of logistic regression analysis are reported in Table 3 [OR (95% CI) and *p*-values in the first and second columns, respectively]. Five variables in the model show *p*-values < 0.05: age at admission, WFNS grade, treatment, days in ICU, and ischemia. The probability of a poor GOS discharge (≤ 3) was five times higher in patients with ischemia compared to those without it [OR (95% CI) 5.02 (2–13.54), *p*-value = 0.001]. In the multivariate logistic model, period (COVID-19 vs. pre-pandemic) was not associated with dichotomized GOS at discharge [OR (95% CI) 2.1 (1–4.48), *p*-value = 0.052]. Additionally, the bivariate analysis, which assessed the association between GOS at discharge and period (COVID-19 vs. pre-pandemic), was not statistically significant if the four patients who tested positive for SARS-CoV-2 were excluded from the COVID-19 period (*p* = 0.072).

**Table 3:** Output of multivariate logistic model (GOS discharge dummy ~ period + age at admission + hypertension + days in intensive care + Fisher grade + WFNS grade + treatment + ischemia + vasospasm)

Variables	OR (95% CI)	*p*-value
Period (COVID-19)	2.1 (1–4.48)	0.052
Age at admission	1.04 (1.01–1.07)	***0.005***
Hypertension (Yes)	1.06 (0.52–2.15)	0.872
Fisher grade	1.05 (0.71–1.55)	0.825
WFNS grade	1.46 (1.15–1.88)	***0.002***
Treatment (None)	9.24 (2.44–46.62)	***0.002***
Days in ICU	1.05 (1.01–1.08)	***0.007***
Ischemia (Yes)	5.02 (2–13.54)	***0.001***
Vasospasm (Yes)	1.92 (0.81–4.61)	0.142
Pseudo *R*^2^	0.30	
AIC	245.8	

In bold and Italics *p*-values < 0.05

## Discussion

This observational multicenter cohort study enrolled 251 patients diagnosed with aSAH in two reference periods. The two subgroups, COVID-19 and pre-pandemic, were quite homogeneous: there were no significant differences in age, gender, previous comorbidities (except for hypertension, which can only be explained by the small sample under examination), WFNS and Fisher at admission, and aneurysm location (Table 1).

From our data, the first relevant issue is a statistically significant diagnostic delay (i.e., time from onset of symptoms to diagnosis and consequent urgent hospitalization) in the COVID-19 period (*p* = 0.030). Both hospital overcrowding [20] and patients’ fear of hospitalization [5, 7, 10, 13, 15, 19, 27] during the pandemic might explain this finding. A delayed access or provision of care has been described for other medical conditions and in many countries during the early phase of COVID-19 pandemic [1, 2, 8, 21, 22, 24, 27–29, 32, 38]. This diagnostic delay, however, did not have negative consequences on outcomes of patients in this study, as shown in Table 2 (*p* = 0.343).

No differences were recorded in the time to treatment in the two periods (*p* = 0.183): there was not a “therapeutic delay” (time between diagnosis and treatment of the aneurysm) during the organization in Hub and Spoke in the COVID-19 period. It can therefore be inferred that, despite the difficulties for patients to reach healthcare facilities or for the healthcare system to manage the pre-hospital emergency, once a diagnosis was obtained, each patient received timely treatment, just as before the pandemic, even if they were transferred from Spoke centers to Hub centers. It is difficult to say what could have happened with a different organization, but considering the amount of resources required by the pandemic, with the conversion of many neurosurgeries into COVID wards, a number of emergencies, including vascular ones, probably could not have been adequately addressed.

ICU and total hospitalization days were significantly less in the COVID-19 period (*p* = 0.023 and 0.016, respectively). This might be explained by either the need of reducing the ICU time as a sign of pressure on the hospital system or as a sign of optimization in high volume centers. The study was not designed to investigate this aspect. As expected, patients who did not undergo treatment, mainly because of poor prognosis at admission, had a significantly worse outcome compared to treated patients.

In the descriptive statistics stratified for period (Table 1), GOS at discharge resulted to be different for the two time periods, mainly due to more patients with GOS 2 and fewer with GOS 5 in the COVID-19 group. Maybe shorter time at the ICU and at the hospital came at a cost after all, but these data could be partly due to shorter follow-up period, as patients were transferred earlier to wards other than neurosurgery or to rehabilitations, where they usually recover after the acute event.

In the multivariate logistic model, age at admission, WFNS grade, days in ICU, and ischemia were prognostic factors for poor outcome (GOS 1–3). The COVID-19 period was slightly significant in the descriptive statistics stratified for GOS (Table 2, *p* = 0.044); this small difference was, however, no longer evident after excluding the 4 patients who were positive for SARS-CoV-2 (*p* = 0.072) and it was not significant in the multivariate logistic model (Table 3, *p* = 0.052). From this point of view, it could be argued that the new organization into a Hub and Spoke system worked adequately: the COVID-19 period itself did not influence outcome of patients with aSAH. This study further confirms that age and poor WFNS at admission, together with ischemia, are negative prognostic factors.

As far as SARS-CoV-2 infection itself is concerned, we could not determine the effect it may have had on SAH outcomes, since during the COVID-19 period, only 4 patients tested positive for SARS-CoV-2. We can, however, observe that all these patients but one had a GOS between 1 and 2. Higher mortality in patients with SAH and COVID-19 compared to those without COVID-19 has already been reported, due to a higher rate of systemic comorbidities such as pulmonary embolism, acute coronary syndrome, and respiratory failure [30, 31]. The negative effects of COVID-19 on mortality and complications have been well documented [14, 17, 25, 34, 36].

## Limitations of the study

Given the high early lethality of aSAH and the statistically significant diagnostic delay that was documented during the COVID-19 period, it is not possible to exclude that some patients died before hospital admission; the study was designed only to investigate hospital admission of aSAH.

## Conclusion

This study documented a significantly increased diagnostic delay for aSAH during the first COVID-19 outbreak in Lombardy, possibly due to patients’ fear of hospitalization. Despite the dramatic situation that the healthcare system was experiencing, the Hub and Spoke organization model, with centralization of neurosurgical emergencies, was not associated with therapeutic delay and, even in the presence of clear signs of system overload, led to results overall comparable to the control period in the management of aSAH.
